# Millisecond newly born pulsars as efficient accelerators of electrons

**DOI:** 10.1038/srep14443

**Published:** 2015-09-25

**Authors:** Zaza Osmanov, Swadesh Mahajan, George Machabeli, Nino Chkheidze

**Affiliations:** 1 School of Physics, Free University of Tbilisi, 0183-Tbilisi, Georgia; 2 Institute for Fusion Studies, The University of Texas at Austin, Austin, Texas 78712; 3 Centre for Theoretical Astrophysics, ITP, Ilia State University, 0162-Tbilisi, Georgia

## Abstract

The newly born millisecond pulsars are investigated as possible energy sources for creating ultra-high energy electrons. The transfer of energy from the star rotation to high energy electrons takes place through the Landau damping of centrifugally driven (via a two stream instability) electrostatic Langmuir waves. Generated in the bulk magnetosphere plasma, such waves grow to high amplitudes, and then damp, very effectively, on relativistic electrons driving them to even higher energies. We show that the rate of transfer of energy is so efficient that no energy losses might affect the mechanism of particle acceleration; the electrons might achieve energies of the order of 10^18^ eV for parameters characteristic of a young star.

One of the most fascinating stories in astro-particle physics is the discovery of high energy cosmic ray (CR) electrons. Two important constituents of this story are: the H.E.S.S (High Energy Stereoscopic System) team’s^1^ claim of the detection of TeV CR electrons of local origin (within ~1 kpc), and the later confirmation of the energy range (.007–1Tev) by the Fermi-LAT collaboration that analyzed the CR electron/positron data samples collected from 4 August 2008 to 4 August 2009^2^.

How do particles acquire such enormous amounts of energy? In astro-particle physics, it is conventional to invoke a Fermi-type process: the stochastic acceleration of particles interacting with strong shocks. Though quite effective, such a mechanism requires a pre acceleration of particles to already high energies^3^. Accelerating light particles like electrons to high energies is even more problematic because the strong synchrotron radiation losses will limit the maximum attainable energy^4^.

One can imagine electrons being boosted to PeV or higher energies if strong energy losses could be, somehow, prevented during the accelerating process. One such mechanism in which the rotation energy of a neutron star was utilized to strongly accelerate particles (via Langmuir waves generated in magnetospheres of pulsars), was recently developed and investigated. It was shown the acceleration process proceeds with almost negligible energy losses^5^. The principle steps in the above mentioned process are:

1. The rotational slow down of a pulsar provides the energy to excite Langmuir waves in the bulk electron-positron plasma in the star atmosphere. All pulsars are characterized by a decreasing rate of spinning, 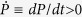, where *P* is the period of rotation.The period is measured by 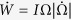, were 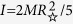 is the moment of inertia of the neutron star, and  g are the pulsar’s mass and the solar mass respectively, 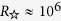 cm is the pulsar’s radius, 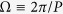 is its angular velocity and 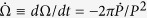.
2. The excited Langmuir waves , then, efficiently damp, preferentially on the much faster but local beam electrons to accelerate them to even larger energies.

We refer the reader to^5^, (and references therein) for the relevant pulsar phenomenology, and details of this acceleration mechanism; henceforth, the latter twould be termed Langmuir-Landau-Centrifugal Drive (LLCD). LLCD is based on the centrifugal acceleration that has been successfully applied to some astrophysical settings^6^,^7^. It is also believed that young pulsars might be sources of ultra high energy cosmic rays^8^. For the paper to be self-sufficient, we will give a synopsis of the theoretical underpinnings of LLCD before applying it to the newly born millisecond pulsars; the results are spectacular; LLCD can drive electron energies all the way to 10^18^ eV.

In this paper, we do not investigate the aftermath of the acceleration era- for instance, the observational patterns caused by the presence of ultra-high energy particles in the magnetospheres of millisecond pulsars. One does expect that electrons with such enormous energies will leave their “fingerprints” on the emission patterns of the corresponding pulsars. The scope of this work could be extended to examine the effects of the electromagnetic radiation that must come from the ultra relativistic electrons; we intend to deal with these problems in near future. Another class of potentially interesting problems (in the context of the present work) are related to the recently discovered events of PeV neutrinos by the Ice Cube experiment. Indeed, electrons and positrons with energies much higher than PeV, might produce PeV neutrinos via several channels: 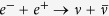; 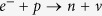.

## Results

The Langmuir-Landau-Centrifugal Drive, derived within the framework of a relatively simple but nontrivial theoretical model, is shown to work highly efficiently in the young millisecond pulsars. LLCD, through a two step process, converts the pulsar spin-down energy into the kinetic energy of electrons. In the first step, the rotation imparted centrifugal particle energy is converted, via a parametrically driven “two stream instability”, to electrostatic Langmuir waves in the bulk electron—positron plasma residing in the pulsar magnetosphere. Landau damping of these centrifugally excited electrostatic waves on the high energy primary beam electrons (beam) transiting through the same region, constitutes the second step.

The linear growth rates of the rotation driven Langmuir instability, calculated for the newly born pulsars, are faster than the typical rotation timescales of particles. The Langmuir instability, thus, is efficient and can rapidly convert the star slow down energy into the electric field energy.

Landau damping of the unstable Langmuir waves on primary beam electrons that converts the electric field energy into particle energy is also shown to be equally rapid. The combination creates a very efficient “machine” that generates ultra high energy (up to EeV) cosmic ray electrons.

We will show that the LLCD mechanism, when applied to newly born fast spinning stars could, indeed, create electron energies that match the highest observed in the CR electrons; we believe that this result has much significance for high energy astro-particle physics.

## Discussion

It is generally believed that to attain the possible maximum possible energy, the gyroradius of the particle should be contained in the acceleration zone. Then, the combination of a strong rotation and a strong magnetic field (of a pulsar) leads to an enormous induced electric field on the light cylinder surface 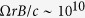 statvolts/cm. This field, might potentially accelerate electrons up to energies of the order of 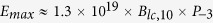 eV^8^, where 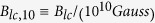, 
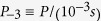
. What one needs , then, is an efficient agency in the rotating magnetosphere that will convert the rotational energy into the acceleration of electrons. We believe that the LLCD does exactly that: the Landau damping of centrifugally induced Langmuir waves guarantees a high efficient channel for acceleration.

For these waves to effectively transfer their energy to particles, the waves phase velocity must be close to the particle speed, which, in this case, approaches the speed of light. Further, in the vicinity of 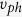, there should be more particles a little slower than the wave than particles which are a little faster (in the opposite situation, the wave will feed off the particles). For the given problem it is always possible to situate 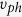 in the desired part of the primary beam spectrum. Since the distribution function decreases with the Lorentz factor, the number of electrons with 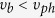 exceeds that of the electrons with 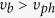, where 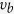 denotes the electron speed. Thus the optimum conditions for effective Landau damping and, therefore, of net energy transfer from the star spin down to electrons, will pertain.

For relativistic plasmas it has been shown that the Landau damping rate is given by^9^


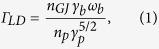


where *n*_*p*_ is the plasma density and 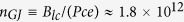 cm^−3^ is the Goldreich-Julian number density. For the primary beam, *γ*_*b*_ and 
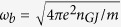
 are, respectively, the Lorentz factor and the plasma frequency. Landau damping is the second step of the acceleration mechanism LLCD.

We now demonstrate the efficiency of LLCD for young millisecond pulsars for which the typical period and slowdown rates are: 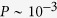 s and 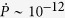 ss^−1^. It is worth noting that, at this stage such pulsars are only, theoretically, predicted^10^; this type of pulsars have not yet been observed.

The efficiency of the LLCD must depend upon the proper coordination of the damping rates on the beam electrons and the growth rates of Langmuir waves in the bulk plasma. In^5^, the magnetospheric plasma was modeled as consisting of two streams with the Lorentz factors *γ*_1,2_ (*γ*_1_ < *γ*_2_). It was shown that the growth rate of the instability is well approximated by the expression


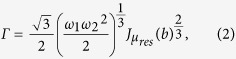


where 
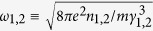
 are the plasma frequencies of the two species, *n*_1,2_ are the corresponding number densities, *J*_*μ*_(*x*) is the Bessel function of the first kind, 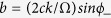, 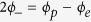 and *ϕ*_*p*_ and *ϕ*_*e*_ are, respectively, the positron’s and electron’s initial phases.

It stands to reason that if the instability growth rate was much greater than the Landau damping rate, there will be little effective energy transfer from waves to the particles. In the diagonally opposite limit with the damping rates far in excess of the growth rates, the waves will not grow much, again resulting in very little transfer from the star rotation to the waves. The most optimum scenario for an overall efficient energy pumping/transfer system, therefore, is realized when the instability growth and Landau damping rates are large (with respect to the kinematic rate, Ω) and comparable, 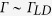. For the two-stream instability, the aforementioned condition can be satisfied for particular combinations of *γ*_1_ and *γ*_2_; for example, for 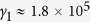, 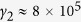.

The total energy gained by the beam particles has been estimated to be^5^


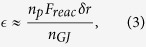


where 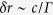, 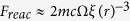, 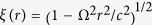 11. From Eq. (3) it is clear that two streams with 
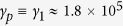
, 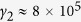, which in turn guarantee the condition, 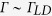, lead to the acceleration of electrons up to energies of the order of 10^18^ eV. This estimate invokes equipartition of energy in the three relevant sp constituents: 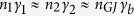 with 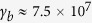, where 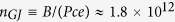 cm^−3^ is the Goldreich-Julian number density^12^ in regions in the vicinity of the light cylinder.

Because the highly relativistic particles will, inevitably, loose energy due to radiation, one must investigate how radiation will affect overall energy transfer. Could radiation, for example, put a stringent limit on the maximum energy acquired?

For highly relativistic electrons and photons with 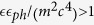 (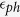 is photon energy), the inverse Compton mechanism operates in the Klein-Nishina (KN) regime^13^. Energy emitted per particle per second is, then, given by the approximate expression 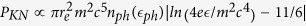
14 where *r*_*e*_ is the classical electron radius. The corresponding time scale of the process is 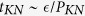. Using the approximate photon number density, 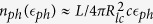, and taking into account the typical values of luminosity in the high energy level, 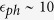 GeV, satisfy *L* > 10^35^ erg/s, it is straightforward to show that for energies of our interest 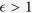 PeV, the aforementioned timescale exceeds the instability time scale by many orders of magnitude. Thus Compton cooling is very slow compared to the wave energy transfer time. The KN time scale goes up with the particle energy implying that at higher energies, the inverse Compton mechanism in the KN channel is too slow to impose any constraints on the maximum attainable electron energy.

The next possible loss mechanism for relativistic particles, moving in magnetic field, is the synchrotron emission with the corresponding estimated power 
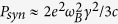
13, where 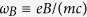 is the cyclotron frequency. According to the standard model of pulsar magnetospheres, there is a significant region over the star’s surface, where the electric field is nonzero (vacuum gap). It is this electric field that, first, accelerates electrons to relativistic energies^15^. One can readily show that the electrons leaving the gap with a 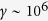 will undergo efficient synchrotron cooling at a short timescale, 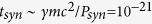 s. Thus, immediately after leaving the gap, the particles radiate their transverse momentum, and very soon transit to the ground Landau state. After that, zipping only along the field lines, the electrons will reach the light cylinder zone in due course of time. It is precisely the region, where the Langmuir waves, always propagating along the field lines, are excited. Therefore the wave interaction with particles will not cause pitch angle scattering, efficiently suppressing the synchrotron mechanism. Quasi linear diffusion, another possible source for imparting a pitch angle^16^, also does not operate because the required condition for diffusion 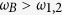, is violated for extremely energetic plasmas (for plasmas with energy density exceeding that of magnetic field). The synchrotron mechanism is not expected to impose any constraints on the maximum attainable energies.

How about the curvature radiation emitted by particles moving along the curved magnetic field lines? To explain force free regime of particles leaving a pulsar’s magnetosphere, we have tried to reconstruct the structure of magnetic field close to the light cylinder surface^17^. We showed that shown that the curvature driven current imparts a toroidal component to the magnetic field. As a result, the field lines lag behind the rotation, gradually erasing the instability. It has been shown that the corresponding timescale is approximately given by


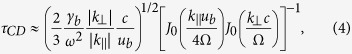


where 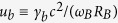 is the curvature drift velocity, 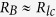 is the curvature radius of a magnetic field line and 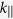 and 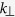 are the components of the wave vector of the induced curvature drift mode respectively along the drift and perpendicular to it. One can see that for a wide range of parameters corresponding to the drift mode with small inclination angles *α* (the angle of the wave vector with respect to the drift direction), the instability timescale is less than the kinematic timescale *P*. In particular, for 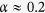 and 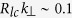, 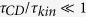. Therefore, the drift instability is so efficient that the magnetic field lines very rapidly reconstruct resulting in a configuration, that enables particles to follow straight line trajectories. The curvature radiation is minimized and does not quite interfere with our energy transfer mechanism.

## Methods

**Figure 1:**
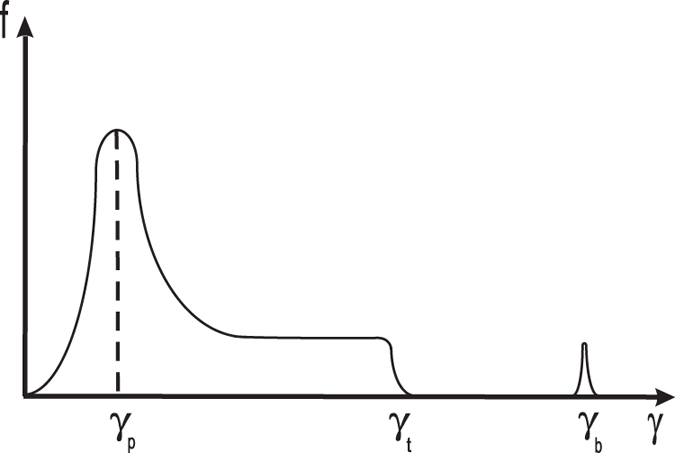
The distribution function versus the Lorentz factor. It is evident that the function consists of two major parts: relatively narrower region with higher Lorentz factors, characterizes the primary Goldreich-Julian beam electrons and the wider part desc ribes the distribution of electrons resulting from the cascade processes of pair creation.

The LLCD was developed using the standard model of the pulsar magnetosphere, where the particle distribution can be, schematically, represented as shown in Fig. 1. The narrower region on the figure describes the primary beam electrons with higher Lorentz factors (*γ*_*b*_). The relatively wider sector with Lorentz factors in the interval 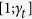 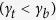 characterizes the secondary particles produced by means of pair creation. Such a shape of the distribution function makes the development of two stream instabilities feasible in the magnetospheres of pulsars^18^. This feature of the electron distributions, along with interesting orbit effects, was exploited in^5^ to demonstrate the the existence of a strong two-stream like instability that is parametrically driven to excite high levels of Langmuir waves.

In the framework of 1 + 1 formalism^19^, the basic system of the Euler equation and continuity equations (for each species) and the Poisson equation, is adequate in describing the centrifugally-excited (rotationally driven) Langmuir waves. The Fourier transformed linear system consists of


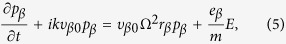



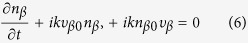



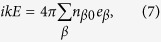


where *β* is the species index (electrons and positrons), *p*_*β*_ and 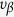 are, respectively, the first order dimensionless momentum 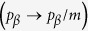 and the zeroth order velocity, *m* is the electron mass, *c* is the speed of light, *e*_*β*_ is the charge of the corresponding particle, *r*_*β*_ is the radial coordinate of the corresponding specie, *n*_*β*_ and *n*_*β*0_ are respectively the perturbed and nonperturbed Fourier components of the density, *k* is the wave number and *E* is the electrostatic field. The first term in the righthand side of the Euler equation comes from the so-called centrifugal force. The centrifugal force, controlled by the following “orbits” of initially relativistic particles 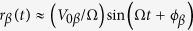, 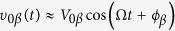
5, where *ϕ* denotes the initial phase of a given species, is time dependent. The resulting Langmuir mode equation is periodic in time (has the generalized Mathieu/Hill form) and exhibits parametric instability.

The time dependent centrifugal force that parametrically drives the electrostatic waves is different for the two species—so are their Lorentz factors.

Since *μ*_*r*_*es*, the order of the Bessel function is rather high, non-zero growth rates will pertain only if the argument *b* is comparable to *μ*_*r*_*es*^20^.

**Figure 2:**
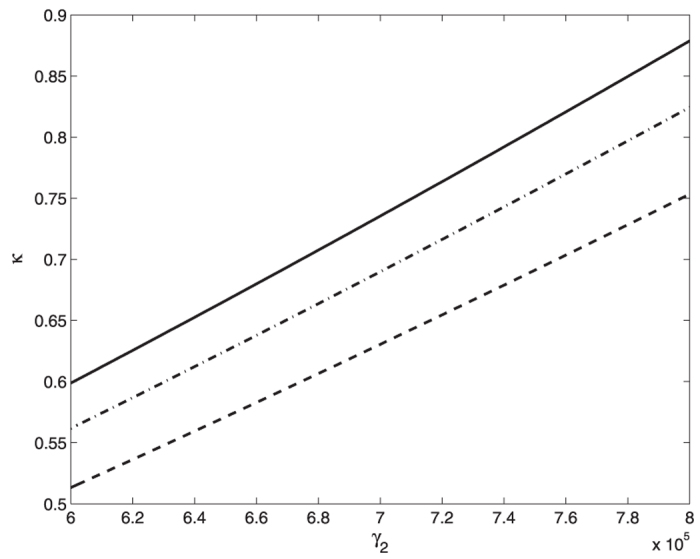
Here we plot the dependence of *κ* on *γ*_2_ for three different values of *γ*_1_: *γ*_1_ = 10^5^ (dashed-dotted line); *γ*_1_ = 1.5 × 10^5^ (dashed line) and *γ*_1_ = 2 × 10^5^ (solid line). As it is clear from the graph, for a quite wide range of Lorentz factors the instability timescale is less than the kinematic timescale, indicating high efficiency of the process.

For the instability to be called “efficient”, the inverse growth time 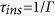 should not exceed the kinematic timescale (also called the escape timescale), 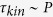. In Fig. 2, we plot the ratio 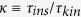 versus *γ*_2_ for different values of *γ*_1_: *γ*_1_ = 10^5^ (dashed-dotted line); *γ*_1_ = 1.5 × 10^5^ (dashed line) and *γ*_1_ = 2 × 10^5^ (solid line). The results are derived for typical young millisecond pulsars with *P* ~ 10^−3^ s and 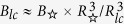, where 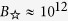 G is the magnetic field close to the pulsar’s surface and 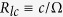 is the light cylinder radius. 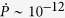 ss^−1^. The condition 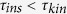 is satisfied for a broad range of Lorentz factors; the instability is, indeed, efficient.

Thus we have reviewed, for the young millisecond pulsars, the first step of LLCD: the existence of an efficient centrifugally excited parametric instability that pumps the spin down energy of the rotator into electrostatic Langmuir waves. Now we review how, through landau damping, this energy will be conveyed to the fastest of the particles. The Landau damping in pulsar magnetospheres has been investigated in^21^, but the authors focus on Alfven waves, but not on the electrostatic waves.

## Additional Information

**How to cite this article**: Osmanov, Z. *et al.* Millisecond newly born pulsars as efficient accelerators of electrons. *Sci. Rep.* **5**, 14443; doi: 10.1038/srep14443 (2015).
